# A dataset of molluscan fauna sampled in river estuaries of medium and small size river in Kyushu island, Japan

**DOI:** 10.3897/BDJ.6.e26101

**Published:** 2018-07-11

**Authors:** Rei Itsukushima, Hiroaki Yoshikawa, Kai Morita

**Affiliations:** 1 Department of Decision Science for a Sustainable Society, Kyushu University, Fukuoka, Japan; 2 Department of Urban and Environmental Engineering, Graduate School of Engineering, Kyushu University, Fukuoka, Japan

**Keywords:** river estuary, molluscan fauna, endangered species, environmental conservation

## Abstract

**Background:**

Many studies have evaluated the ecological integrity of large-scale estuaries of continental rivers using biotic indicators such as fish, phytoplankton and benthic communities. However, few studies have focused on the river estuaries of small and medium rivers. Molluscan fauna data in large estuaries or in the estuaries of large rivers have been collected by the The National Census on River Environments (conducted by the Ministry of Land, Infrastructure, Transport and Tourism) or National Survey on the Natural Environment (conducted by the Ministry of Environment). On the other hand, molluscan fauna of small and medium rivers are managed by local governments and have rarely been investigated.

**New information:**

This paper provides basic information on the molluscan fauna of 70 rivers in Kyushu, Japan, collected with the aim of conserving estuaries of small and medium rivers. In total, 37 families, 82 species and 21,827 individuals were collected. The data are all accessible from the document “A dataset of shellfish fauna sampled in estuaries of medium and small rivers in Kyushu, Japan (http://ipt.pensoft.net/resource.do?r=shellfishes_in_kyushu)”. According to the Red Data Book published by the Japanese Ministry of Environment in 2018, 3 species were determined as Critically endangered and Endangered, 6 species were determined as Vulnerable and 13 species were determined as Near Threatened. The proportions of individuals classified as Critically endangered and Endangered from the total number of individuals were extremely low, but the proportions of Near Threatened individuals were high. Our results indicate that the risk of molluscan extinction in small- and medium-sized river estuaries in Kyushu is high and that immediate conservation is necessary.

## Introduction

Estuaries are located at the boundary between the land and sea and present particular environments that constantly fluctuate with the periodic function of waves or tides (Dyer 1997, Schröder-Adams et al. 2014). Estuaries provide multiple ecosystem services such as nutrient cycling, mitigation of climate change and habitat provision (Carpenter et al. 2009). However, because of the abundance of biodiversity, land nutrients and natural resources, human beings have exploited estuaries for long periods of time and the anthropogenic impact on estuaries is very high (Cohen 1998, Edgar et al. 2000). Anthropogenic impact causes degradation of water quality, disappearance of habitat and decreasing natural resources (McIntyre 1995, Kennish 2002, Howarth 2008). Many studies have been conducted using biotic indicators, such as fish fauna (Albouy et al. 2011, Halpern and Floeter 2008, Reserarits and Chalcraft 2007, Silva-Júnior et al. 2017), phytoplankton communities (Cloern and Dufford 2005, Paerl et al. 2007, Bhattacharjee et al. 2013, Roshith et al. 2018) and benthic communities (Alexandridis et al. 2017, Kristensen et al. 2014, Martin et al. 2010), mainly on the large-scale estuaries of continental rivers. However, there are very few studies which have focused on the estuaries of small and medium rivers.

In this paper, we investigated molluscan fauna to provide basic information for the environmental conservation of estuaries of small and medium rivers. The river estuaries assessed in this study constitute environments with large environmental gradients, in which water quality, riverbank sediment material and micro-topography change in the longitudinal and transverse sections of the river (Kusuda and Yamamoto 2008). Molluscan fauna data in large estuaries or in the estuaries of large rivers have been collected by the The National Census on River Environments (conducted by the Ministry of Land, Infrastructure, Transport and Tourism) or National Survey on the Natural Environment (conducted by the Ministry of Environment). On the other hand, molluscan fauna of small and medium rivers are managed by local governments and have rarely been investigated. In addition, studies evaluating estuarine habitats in small and medium river estuaries have focused primarily on fish and crustaceans, but brackish fish accidentally invade river estuaries. Therefore, the relationship between the habitat and occurrence of brackish fish remains unclear. There is a possibility that brackish fish constitute poor indicator species.

We selected molluscan fauna to evaluate the integrity of river estuaries. Molluscan fauna respond sensitively to water quality and bottom sediments and include species that inhabit only one particular environment or have a low capacity to thrive in different habitats. Molluscan species at individual locations directly reflect the environmental conditions at these sites (Sato 2011). Therefore, molluscan species are ideal for evaluating the environmental conditions and determining the impact of human activities on estuarine environments (Blanchet et al. 2014, Koutsoubas 2000).

In this paper, we report data of molluscan fauna collected in 70 rivers in Kyushu, Japan with the aim of providing information for the conservation of estuaries of small and medium rivers.

## Sampling methods

### Study extent

We defined the land of the low flow channel as a habitat and set one to three sampling points at each habitat according to the habitat area. Eight kinds of habitats were set from the viewpoint of particle size of the sediment, vegetation and artificial structure (silt, sand, gravel, boulder, bedrock, riprap, concrete construct and vegetation). Habitats at mid tide and spring tide belonging to one reach section (approximately 10 times the width of the river) were selected as investigation sites. At each habitat, we identified the species after collecting molluscan fauna (*Bivalvia*, *Gastropoda* and *Polyplacophora*) from the surface layer and from10-cm deep in the ground with 50-cm square quadrats. 3–15 quadrats were investigated in each site.

## Geographic coverage

### Description

**Figure 1.**
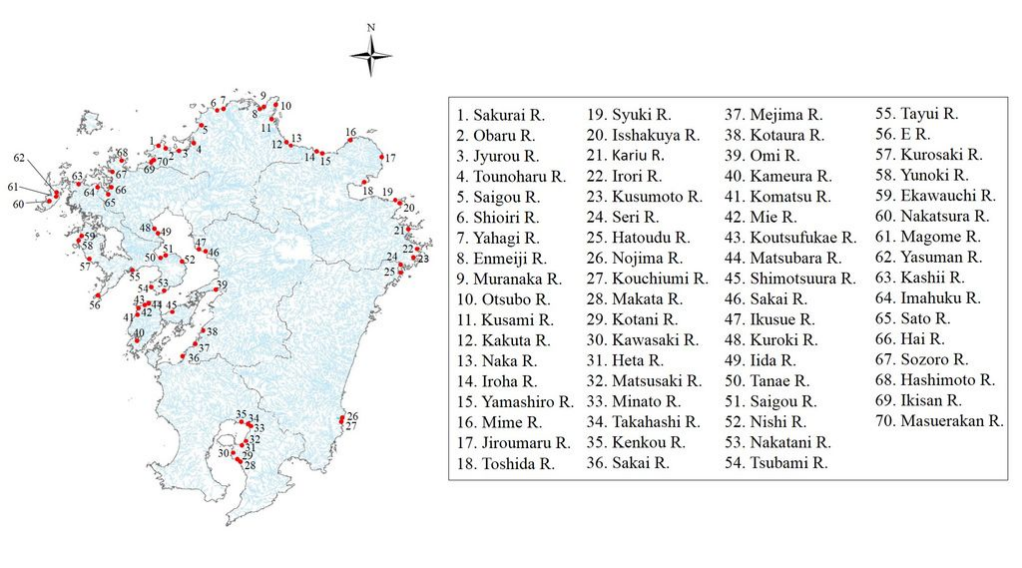
Locations of study area.

We surveyed 70 river estuaries in the Kyushu region in Japan (Fig. 1). Watershed areas of investigated rivers ranged from 1 km^2^ to 60 km^2^. The rivers were selected based on the variation in environmental conditions, such as the Ariake Sea and Buzen Sea, where the tide range is large and the rivers flowing to the Sea of Japan, where the wave energy is dominant.

### Coordinates

31.006 and 33.925 Latitude; 129.54 and 132.012 Longitude.

## Taxonomic coverage

### Description

**Figure 2.**
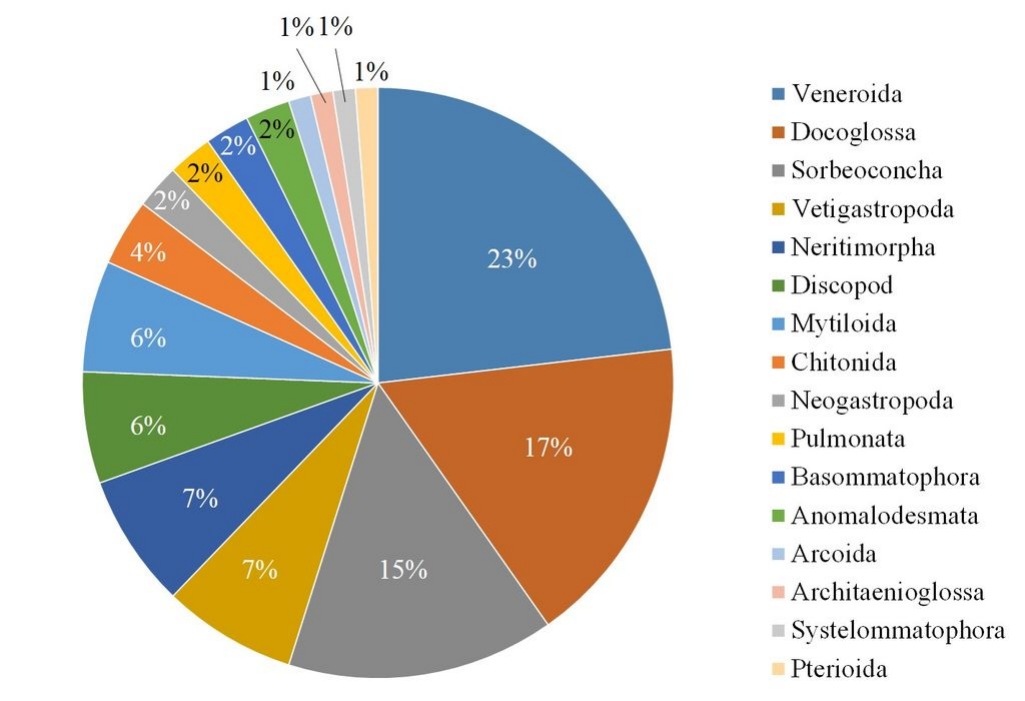
Taxonomic coverage (by taxonomic order).

**Figure 3.**
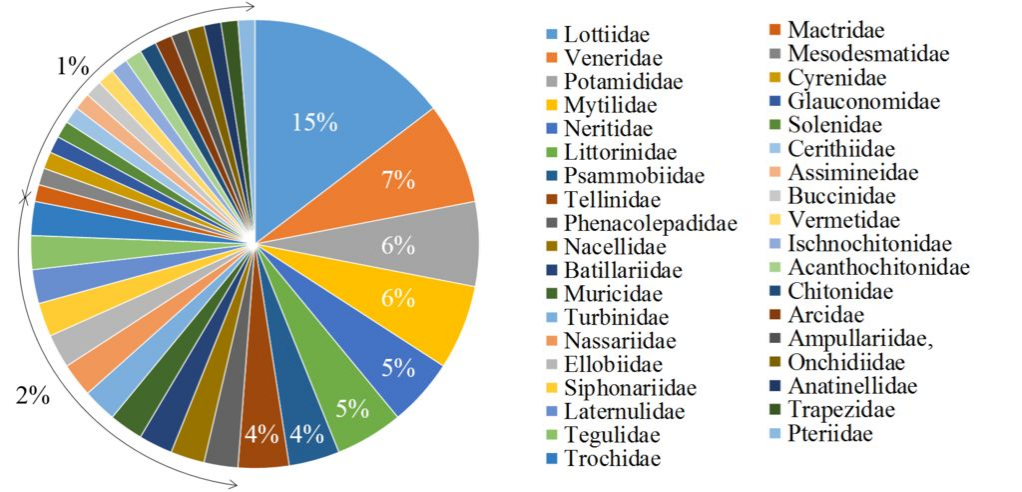
Taxonomic coverage (by taxonomic family).

The orders are Veneroida (19 species), Docoglossa (14 species), Sorbeoconcha (12 species), Vetigastropoda (6 species), Neritimorpha (5 species), Discopod (5 species), Mytiloida (5 species), Chitonida (3 species), Neogastropoda (2 species), Pulmonata (2 species), Basommatophora (2 species), Anomalodesmata (2 species), Arcoida (1 species), Architaenioglossa (1 species), Systelommatophora (1 species) and Pterioida (1 species) (Fig. 2). We recorded species in the families Lottiidae (12 species), Veneridae (6 species), Potamididae (5 species), Mytilidae (5 species), Neritidae (4 species), Littorinidae (4 species), Psammobiidae (3 species), Tellinidae (3 species), Phenacolepadidae (2 species), Nacellidae (2 species), Batillariidae (2 species), Muricidae (2 species), Turbinidae (2 species), Nassariidae (2 species), Ellobiidae (2 species), Siphonariidae (2 species), Laternulidae (2 species), Tegulidae (2 species), Trochidae (2 species), Mactridae (1 species), Mesodesmatidae (1 species), Cyrenidae (1 species), Glauconomidae (1 species), Solenidae (1 species), Cerithiidae (1 species), Assimineidae (1 species), Buccinidae (1 species), Vermetidae (1 species), Ischnochitonidae (1 species), Acanthochitonidae (1 species), Chitonidae (1 species), Arcidae (1 species), Ampullariidae (1 species), Onchidiidae (1 species), Anatinellidae (1 species), Trapezidae (1 species) and Pteriidae (1 species) (Fig. 3).

## Temporal coverage

### Notes

The survey was conducted from 28 April 2015 to 23 November 2017.

## Usage rights

### Use license

Creative Commons Public Domain Waiver (CC-Zero)

## Data resources

### Data package title

A dataset of shellfish fauna sampled in estuaries of medium and small rivers in Kyushu, Japan

### Resource link

http://ipt.pensoft.net/resource.do?r=shellfishes_in_kyushu

### Number of data sets

1

### Data set 1.

#### Data set name

A dataset of molluscan fauna sampled in estuaries of medium and small rivers in Kyushu, Japan

#### Number of columns

30

#### 

**Data set 1.** 

Column label	Column description
occurrenceID	An identifier for the Occurrence.
basisOfRecord	The specific nature of the data record.
eventDate	The date-time or interval during which an Event occurred.
scientificName	The full scientific name.
kingdom	The full scientific name of the kingdom in which the taxon is classified.
phylum	The full scientific name of the phylum or division in which the taxon is classified.
class	The full scientific name of the class in which the taxon is classified.
order	The full scientific name of the order in which the taxon is classified.
family	The full scientific name of the family in which the taxon is classified.
taxonRank	The taxonomic rank of the most specific name in the scientificName as it appears in the original record.
identifiedBy	A list (concatenated and separated) of names of people, groups or organisations who assigned the Taxon to the subject.
decimalLatitude	The geographic latitude (in decimal degrees, using the spatial reference system given in geodeticDatum) of the geographic centre of a Location.
decimalLongitude	The geographic longitude (in decimal degrees, using the spatial reference system given in geodeticDatum) of the geographic centre of a Location.
geodeticDatum	The ellipsoid, geodetic datum or spatial reference system (SRS) upon which the geographic coordinates given in decimalLatitude and decimalLongitude as based.
countryCode	The standard code for the country in which the Location occurs. Recommended best practice is to use ISO 3166-1-alpha-2 country codes.
individualCount	The number of individuals represented present at the time of the Occurrence.
organismQuantity	A number or enumeration value for the quantity of organisms.
organismQuantityType	The type of quantification system used for the quantity of organisms.
habitat	A category or description of the habitat in which the Event occurred.
catalogNumber	A list (concatenated and separated) of previous or alternate fully qualified catalogue numbers or other human-used identifiers for the same Occurrence, whether in the current or any other data set or collection.
language	A language of the resource. Recommended best practice is to use a controlled vocabulary such as RFC 4646 [RFC4646]
country	The name of the country or major administrative unit in which the Location occurs. Recommended best practice is to use a controlled vocabulary such as the Getty Thesaurus of Geographic Names.
stateProvince	The name of the next smallest administrative region than country (state, province, canton, department, region etc.) in which the Location occurs.
municipality	The full, unabbreviated name of the next smallest administrative region than county (city, municipality etc.) in which the Location occurs. Do not use this term for a nearby named place that does not contain the actual location.
locality	The specific description of the place. Less specific geographic information can be provided in other geographic terms (higherGeography, continent, country, stateProvince, county, municipality, waterBody, island, islandGroup). This term may contain information modified from the original to correct perceived errors or standardise the description.
modified	The most recent date-time on which the resource was changed. For Darwin Core, recommended best practice is to use an encoding scheme, such as ISO 8601:2004(E).
year	The four-digit year in which the Event occurred, according to the Common Era Calendar.
month	The ordinal month in which the Event occurred.
day	The integer day of the month on which the Event occurred.
locationID	An identifier for the set of location information (data associated with dcterms:Location). May be a global unique identifier or an identifier specific to the data set.

## Additional information

Of the molluscan fauna that occurred in each river, we focused on the endangered species described in the Red List (Japanese Ministry of the Environment 2018). Simple environmental evaluation of the target river was conducted using species registered as Critically endangered and Endangered (CR + EN), Vulnerable (VU) and Near Threatened (NT) in the Red List. We assessed species composition by threatened status for rivers where more than 5 threatened species appeared. We also assessed population structure (i.e. number of individuals) by threatened status for rivers where more than 5 threatened species occurred.

According to the Red Data Book published by the Japanese Ministry of Environment in 2018, *Cerithidea largillierti*, *C. ornata* and *Laternula boschasina* were listed as CR + EN. *Cerithium coralium*, *Pirenella alata*, *Ellobium chinense*, *Moerella iridescens*, *Glauconome chinensis* and *Meretrix lusoria* were classified as VU. *Neripteron cornucopia*, *Phenacolepas pulchella*, *Batillaria multiformis*, *C. rhizophorarum*, *C. cingulata*, *Laemodonta exaratoides*, *Coecella chinensis*, *Nitidotellina hokkaidoensis*, *Moerella rutila*, *Psammotaea minor*, *Trapezium liratum*, *Corbicula javanica* and *Anomalodiscus squamosus* were classified as NT.

**Figure 4.**
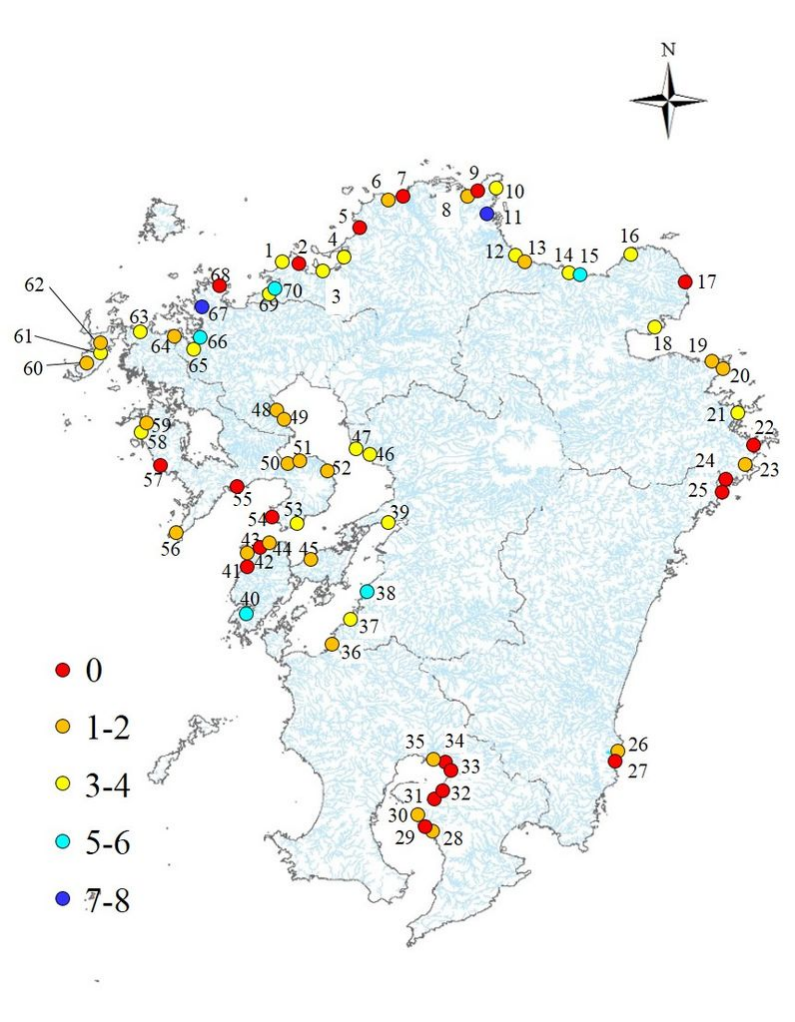
Geographical distribution of threatened species.

**Figure 5.**
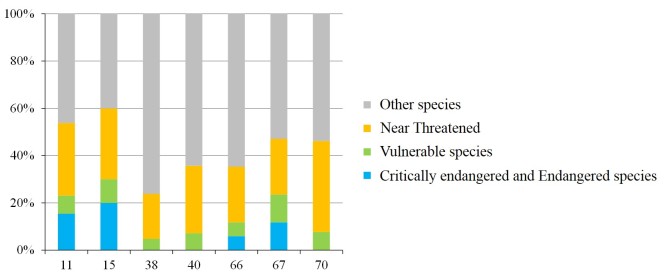
Species composition in rivers with more than 5 threatened species.

**Figure 6.**
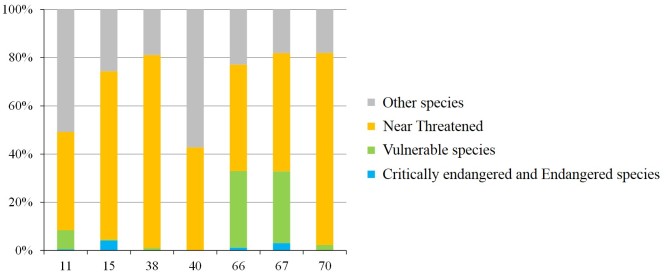
Population structure (i.e. numbers of individuals) in rivers with more than 5 threatened species.

The occurrence of endangered species differed amongst rivers (Fig. 4); the proportion of threatened species exceeding 50% in the Kusami and Yamashiro Rivers (Fig. 5). The proportion of threatened species was approximately 20% in the Kotaura River. The proportions of CR + EN species were low in most rivers and the highest (20%) in the Yamashiro River (Fig. 5). Over 40% of individuals belonged to threatened species in all rivers (Fig. 5). In the Yamashiro, Kotaura, Sozoro and Masuerakan Rivers, approximately 80% of occurrences were of individuals classified as endangered (Fig. 6). In the Sozoro River and the Hai Rivers, 30% of recorded individuals belonged to *Pirenella alata*, a VU species. On the other hand, the proportion of species classified as CR + EN in the total number of individuals was extremely low (Fig. 6).

We found threatened species in 50 out of the 70 target rivers with important implications for the conservation of estuarine species and environments. The number of threatened species was the highest in the Sozoro River (8 species) located in Imari Bay, followed by the Kusami River (7 species) flowing into the Buzen Sea. Many endangered species were found in rivers flowing into large-scale bays with high tide ranges such as the Buzen Sea, the Ariake Sea and the Yatsushiro Sea. There were few threatened species in river estuaries influenced by high wave energy flowing into the open seas such as the Sea of Genkai and the Sea of Hyuganada. This may be explained by the large tidelands formed in bays with large tidal differences, which potentially lead to high species diversity. In contrast, in river estuaries influenced by large wave energies, the diversity of the physical environment is lower than that of tidal flats and this seems to affect the number of endangered species. Therefore, consideration of the potential physical environment formed by external forces acting on the river estuaries is essential when evaluating biota for environmental conservation and restoration. Our results indicate that the risk of molluscan extinction in Kyushu is high and that immediate conservation is necessary.

Images of the specimen was presented (Suppl. material 1). A simple list of the taxa included in the study describing family, genus, species author and date was also presented (Suppl. material 2).

## Supplementary Material

Supplementary material 1 Images of specimen Data type: images
Brief description: images of specimen
File: oo_212074.pdf Rei Itsukushima

Supplementary material 2 Sepecies list Data type: occurences
Brief description: simple sepecies list
File: oo_212077.xlsx Rei Itsukushima
