# Supplementary material for: A dataset of molluscan fauna sampled in river estuaries of medium and small size river in Kyushu island, Japan
Source: Biodivers Data J. 2018 Jul 11;(6):e26101. doi: 10.3897/BDJ.6.e26101 (PMC6052029; doi:10.3897/BDJ.6.e26101)
Supplement: Supplementary material 1 — Images of specimen [file bdj-06-e26101-s001.pdf]

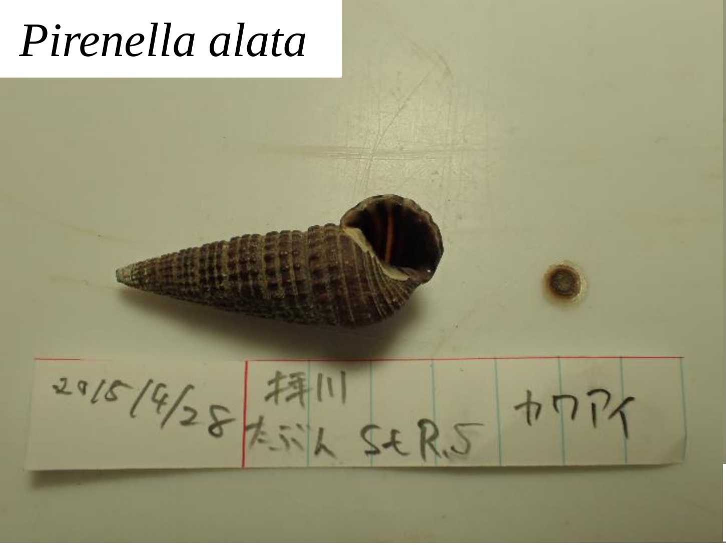

Pirenella alata

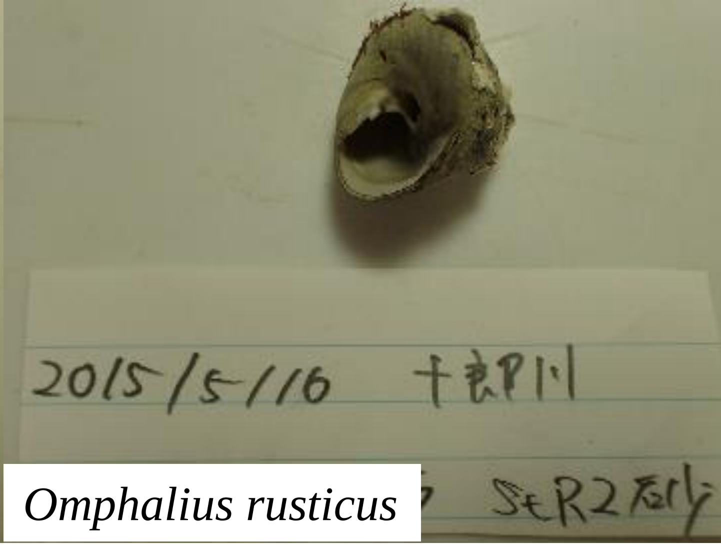

Omphalius rusticus

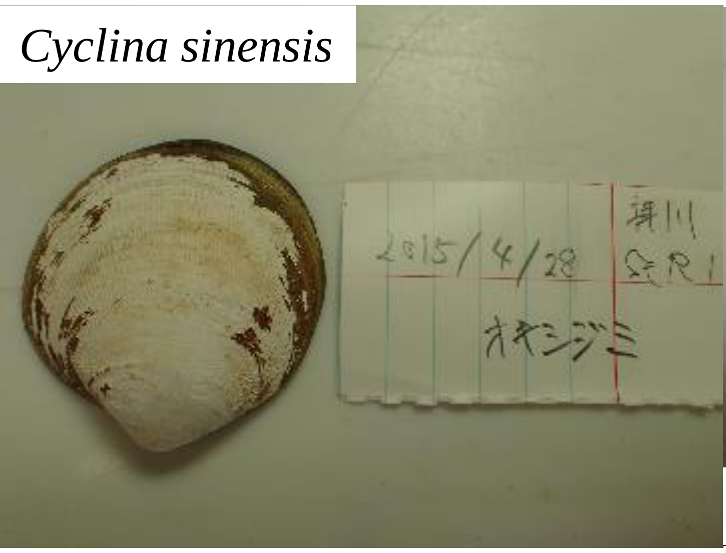

Cyclina sinensis

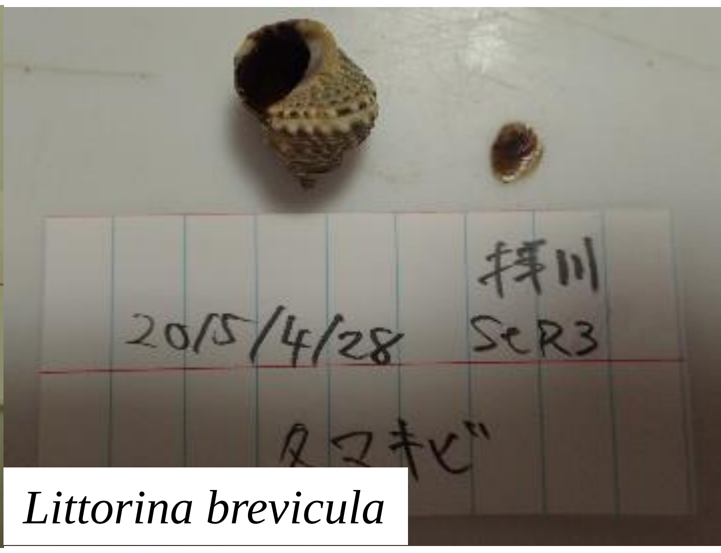

Littorina brevicula

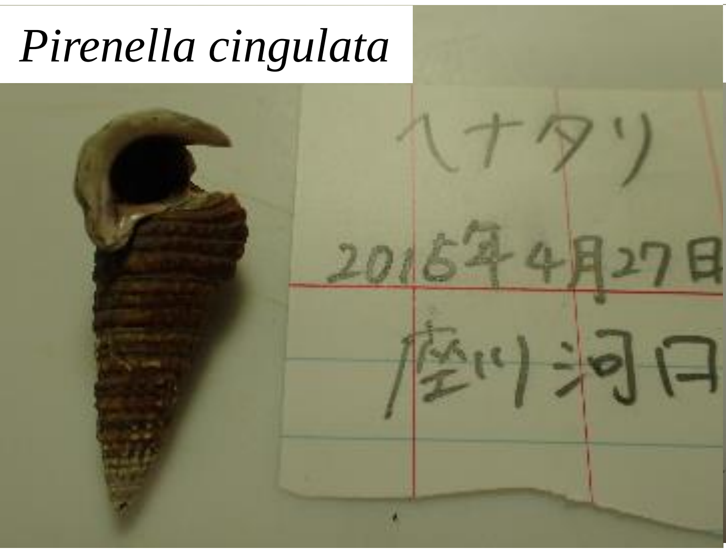

Pirenella cingulata

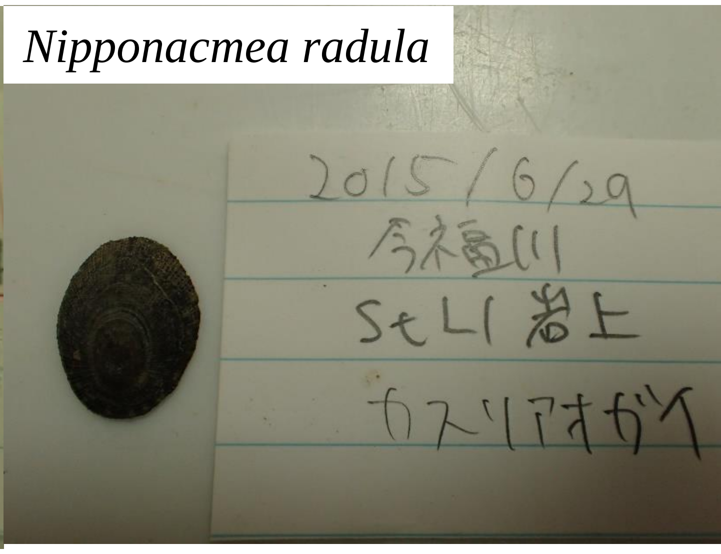

Nipponacmea radula

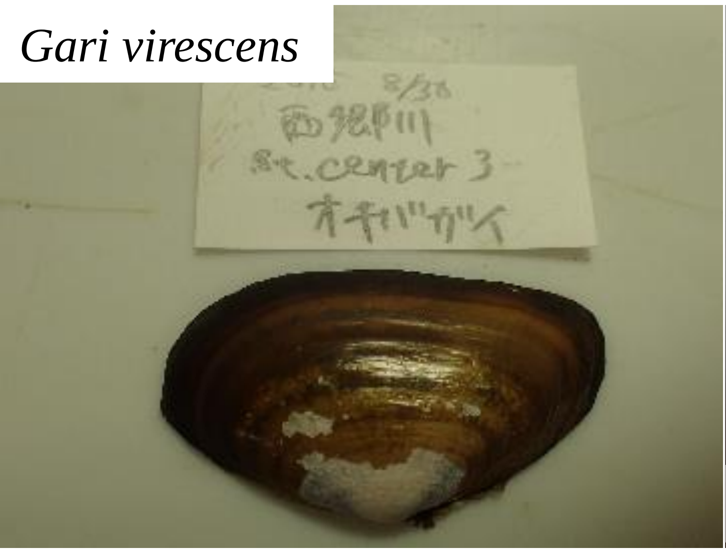

Gari virescens

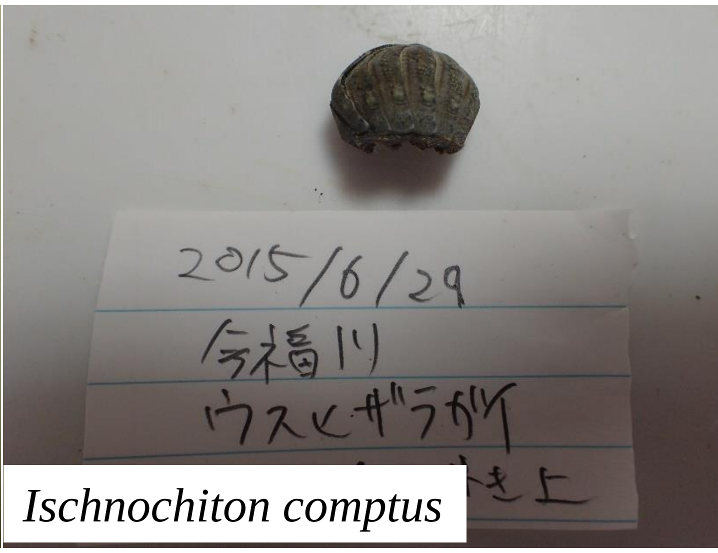

Ischnochiton comptus

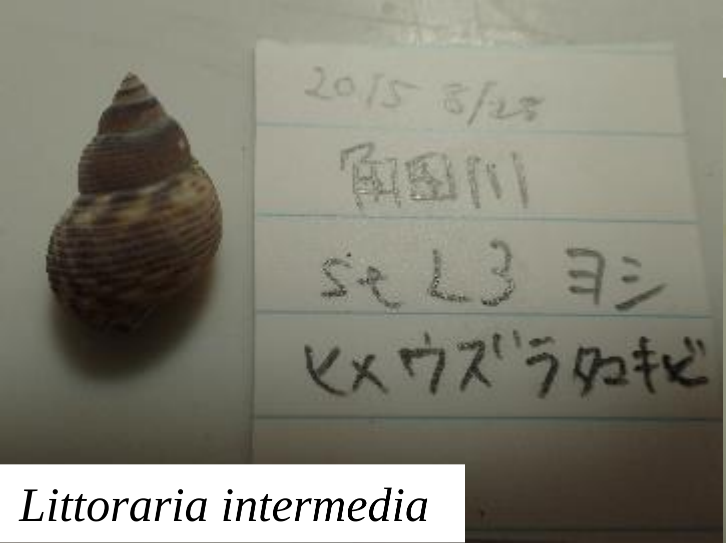

Littoraria intermedia

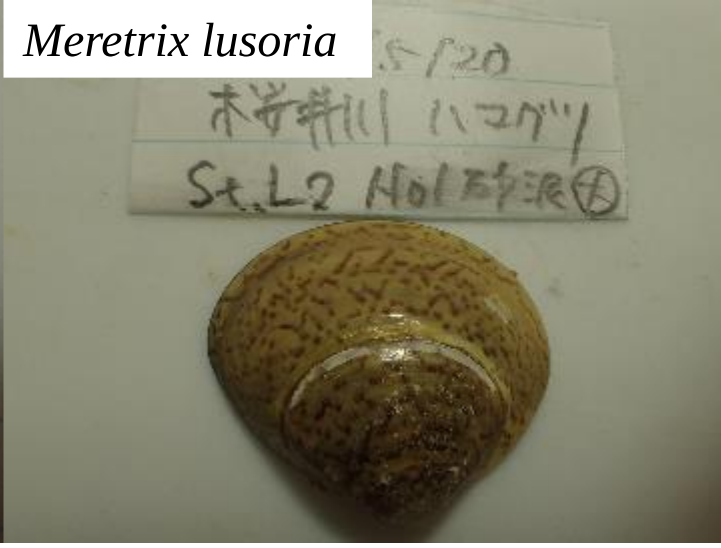

Meretrix lusoria

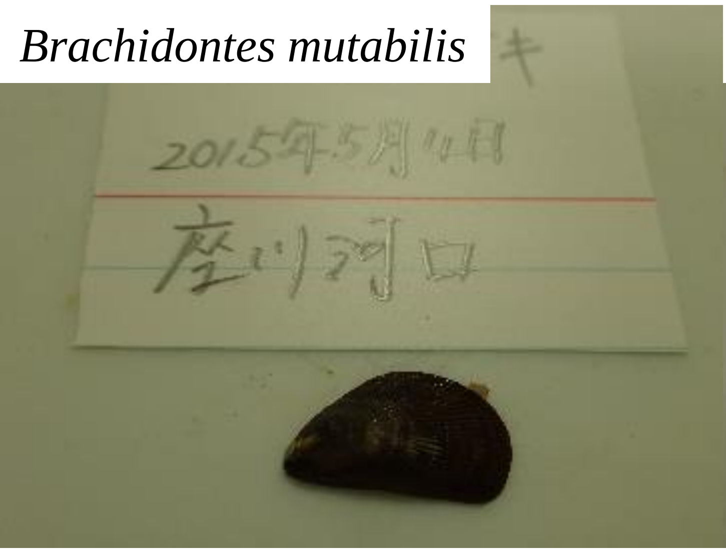

Brachidontes mutabilis

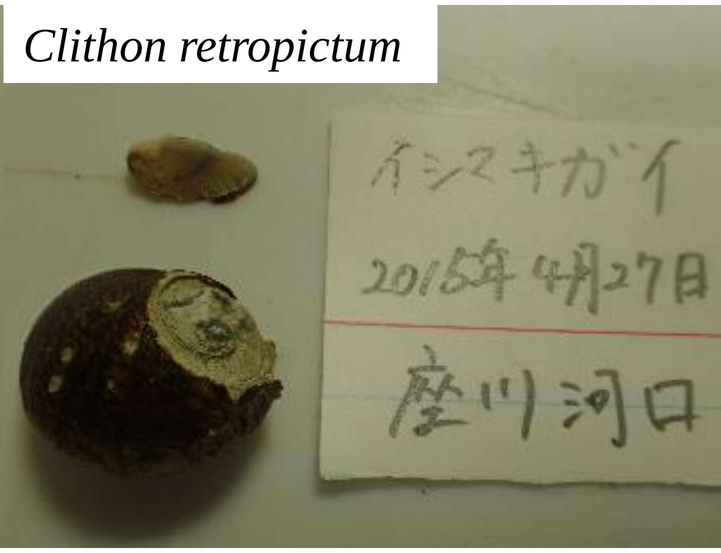

Clithon retropictum

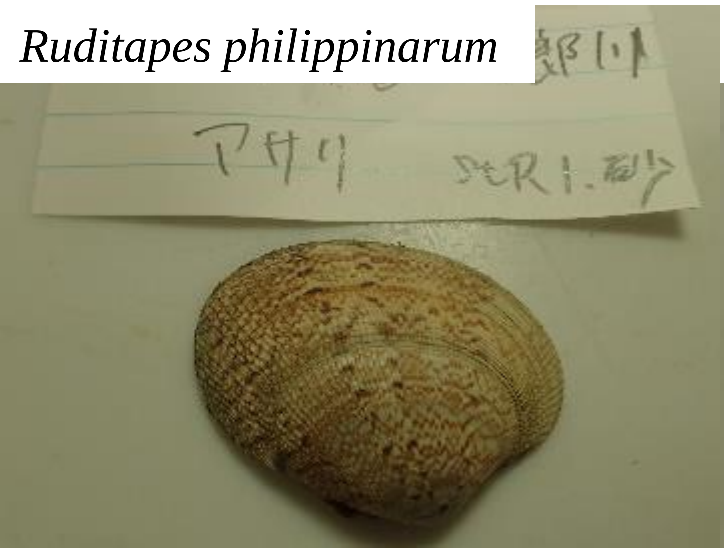

Ruditapes philippinarum

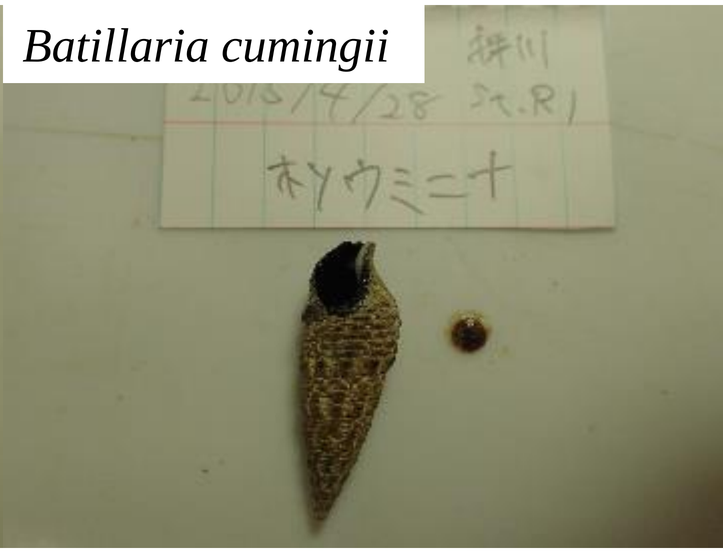

Batillaria cumingii

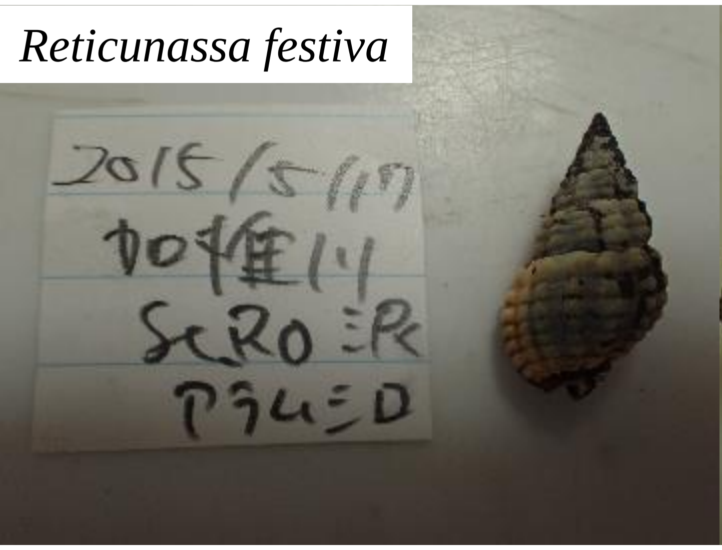

Reticunassa festiva

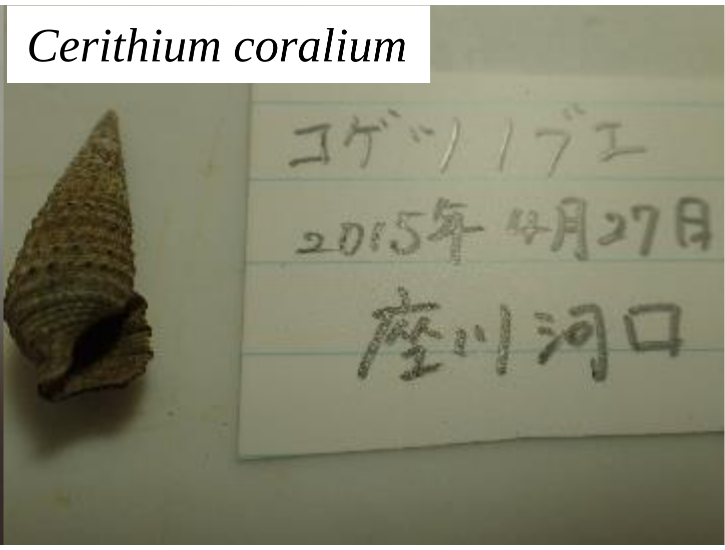

Cerithium coralium

Batillaria multiformis

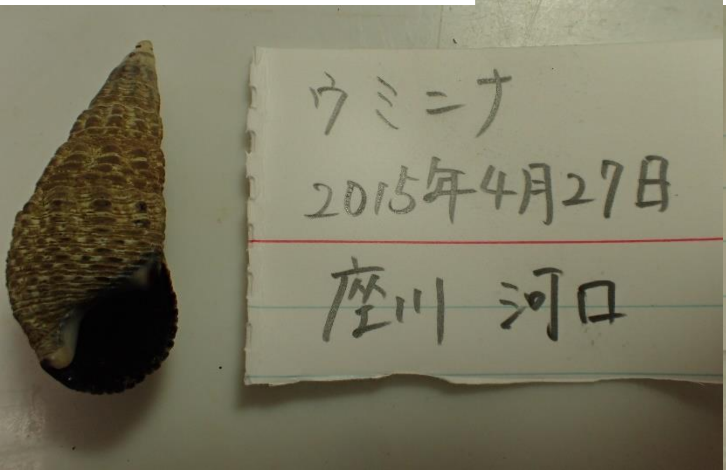

Assiminea sp.

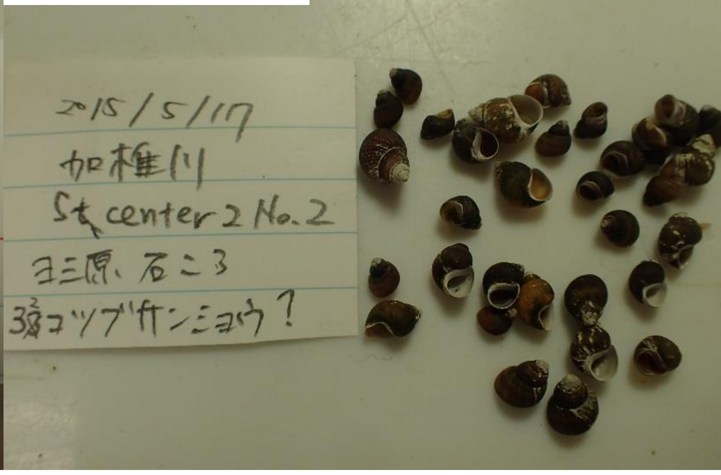

Cerithidea rhizophorarum

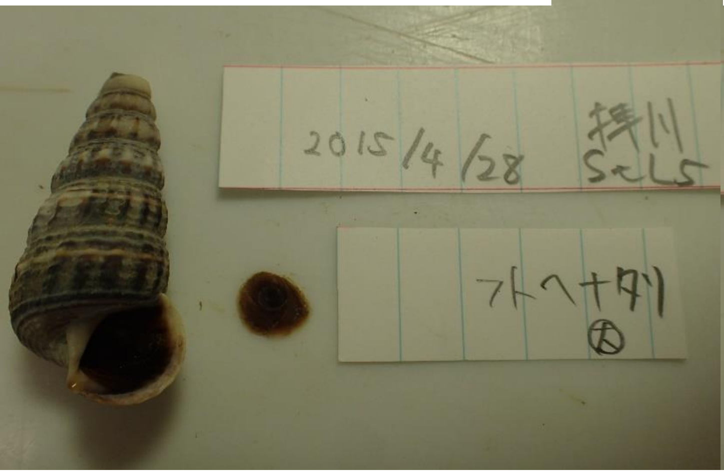

Cerithidea balteata

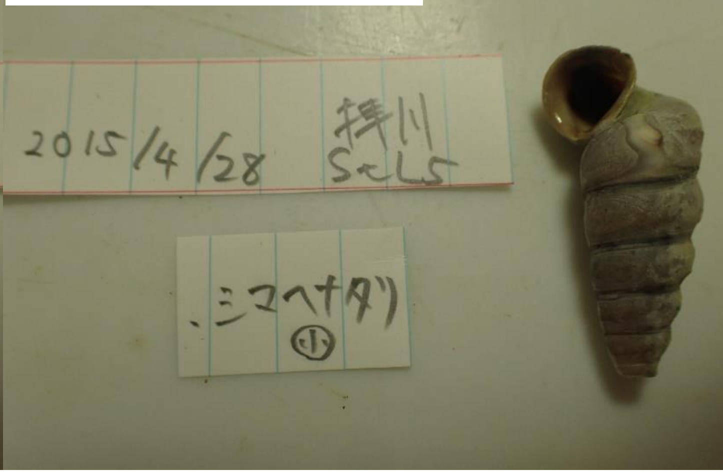

Cellana nigrolineata

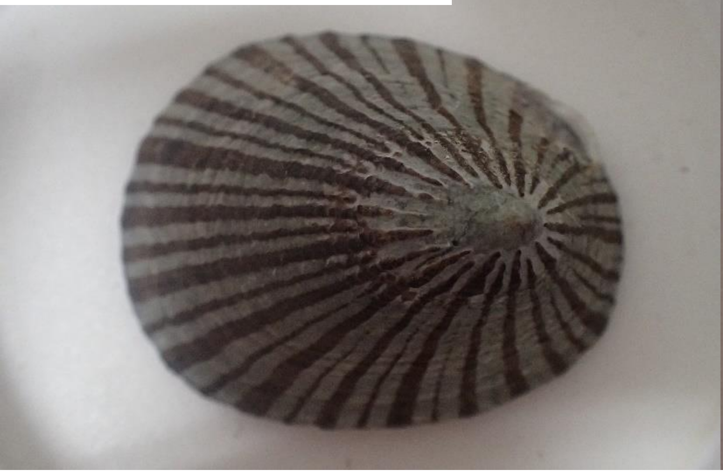

Littoraria articulata

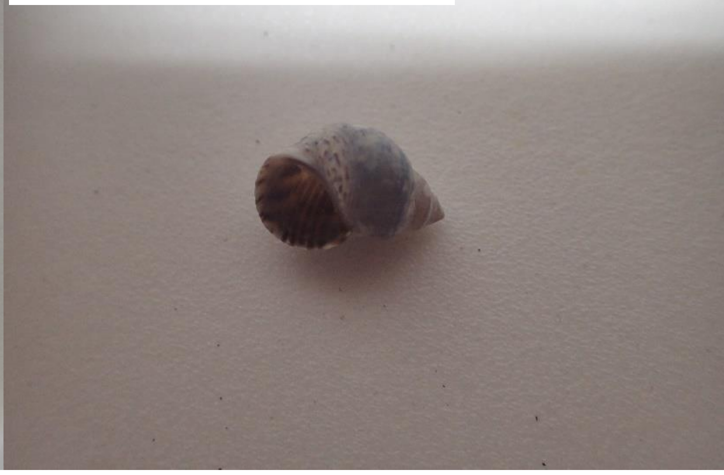

Phenacolepas pulchella

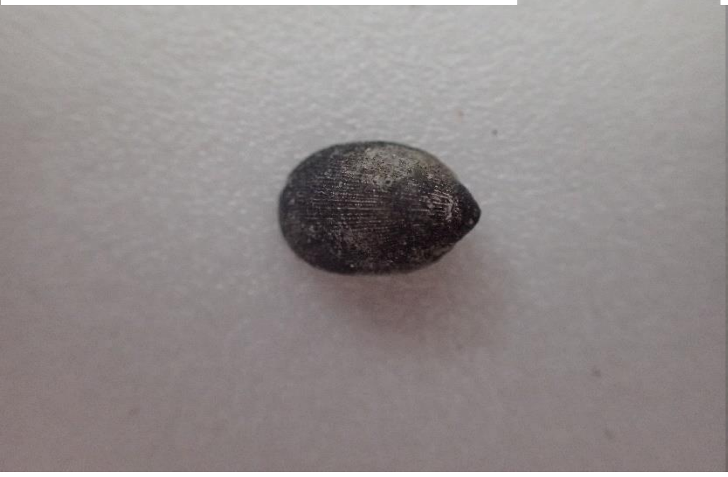

Nipponacmea schrenckii

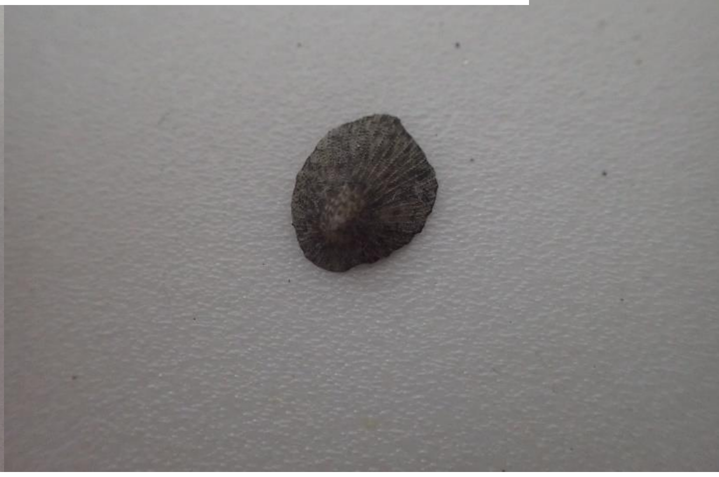

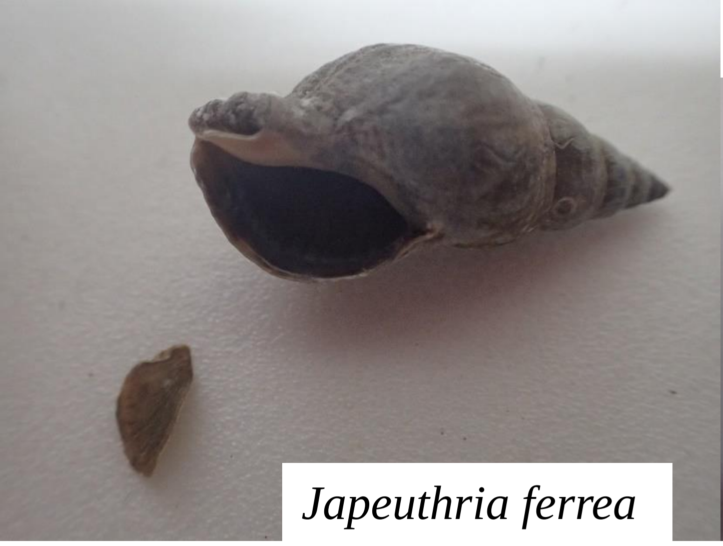

Japeuthria ferrea

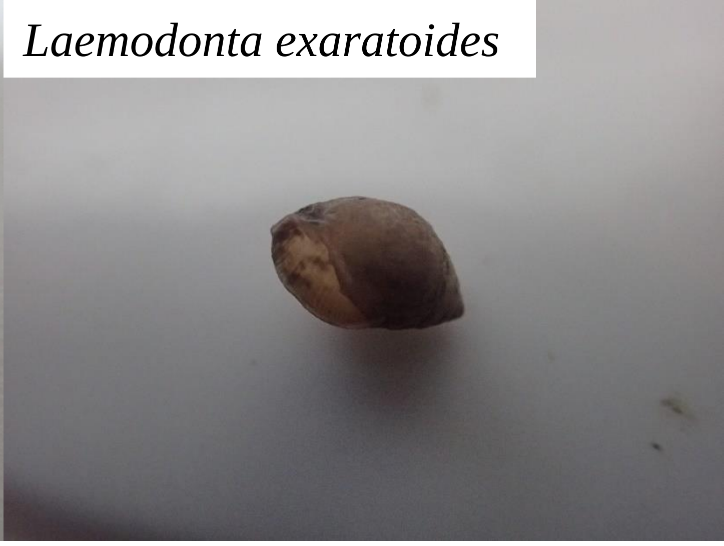

Laemodonta exaratoides

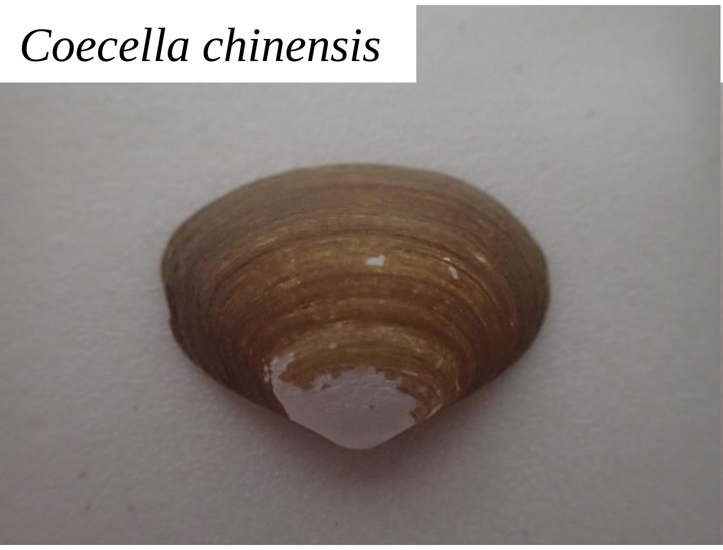

Coecella chinensis

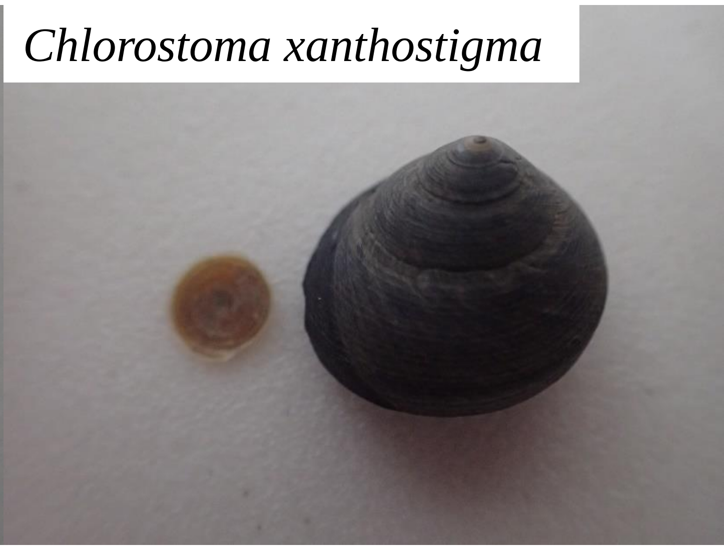

Chlorostoma xanthostigma

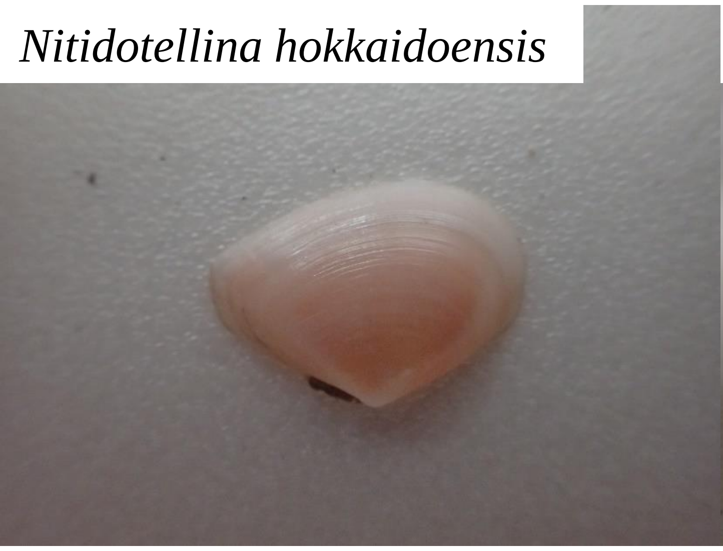

Nitidotellina hokkaidoensis

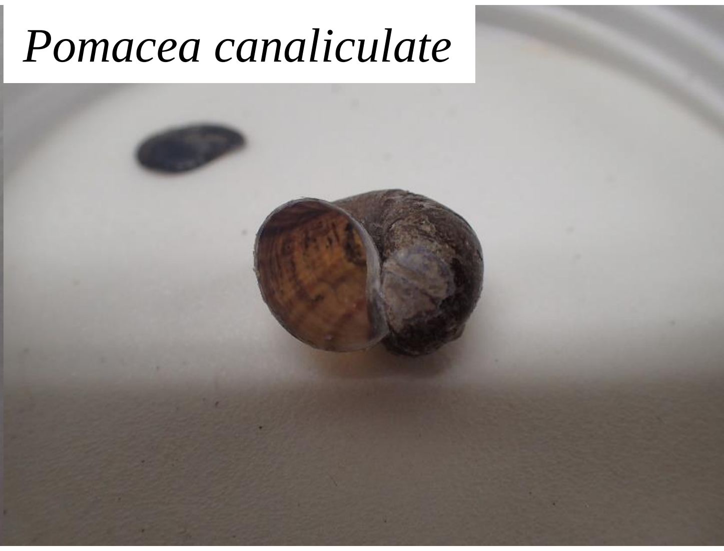

Pomacea canaliculate

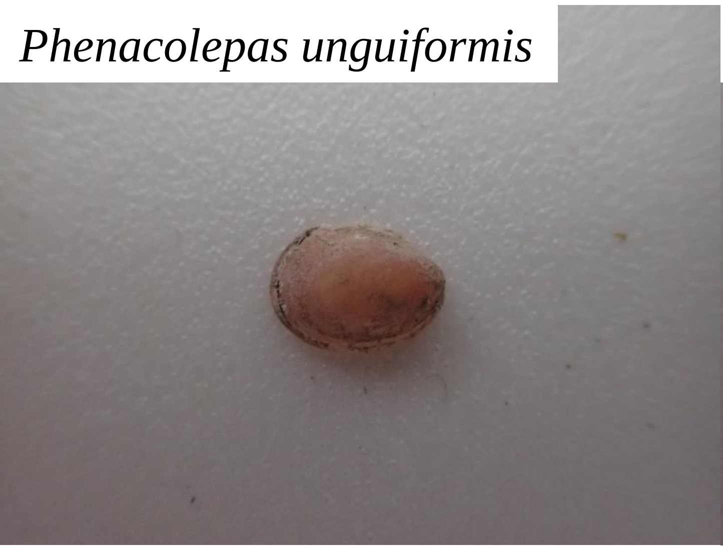

Phenacolepas unguiformis

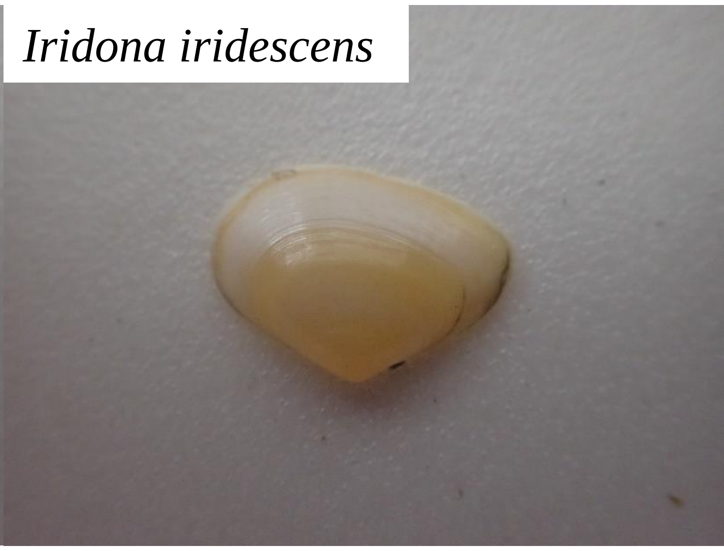

Iridona iridescens

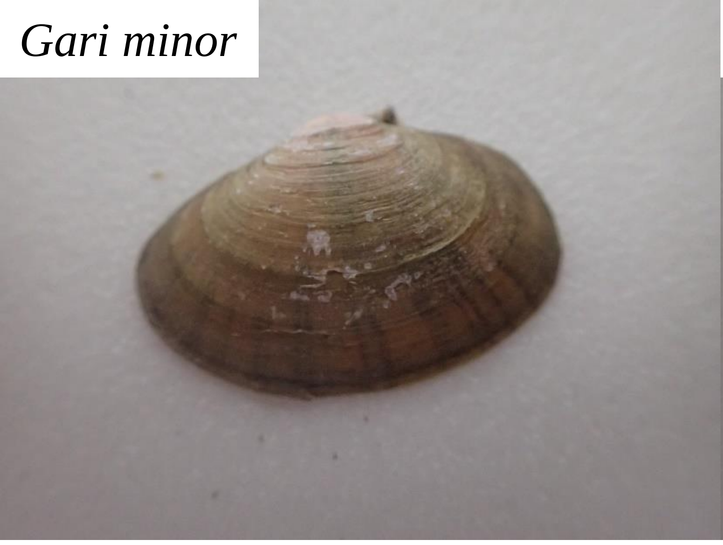

Gari minor

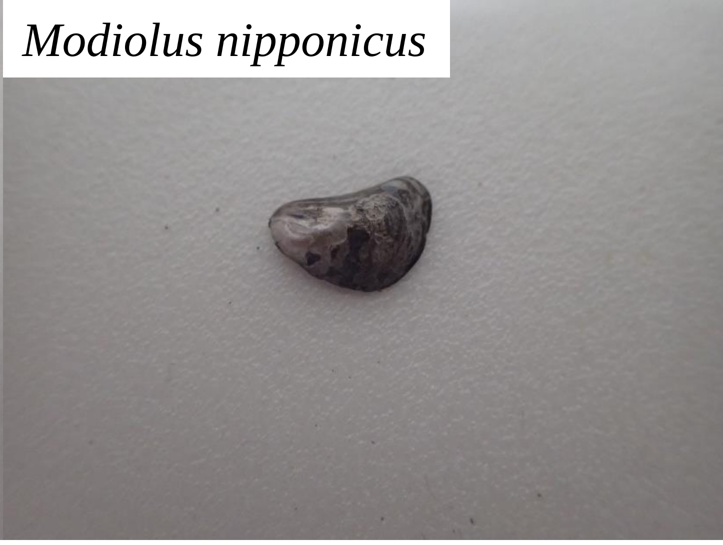

Modiolus nipponicus

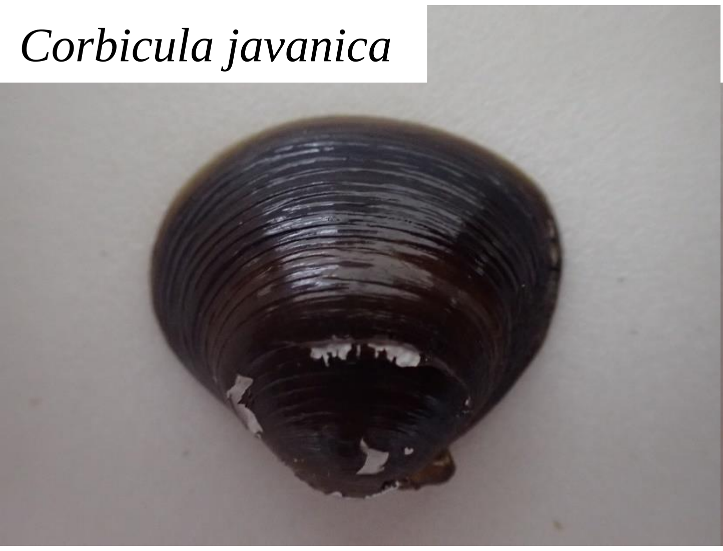

Corbicula javanica

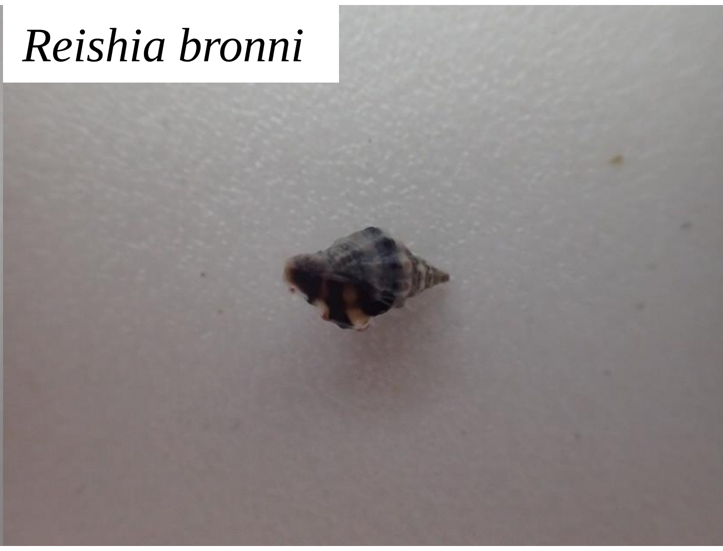

Reishia bronni

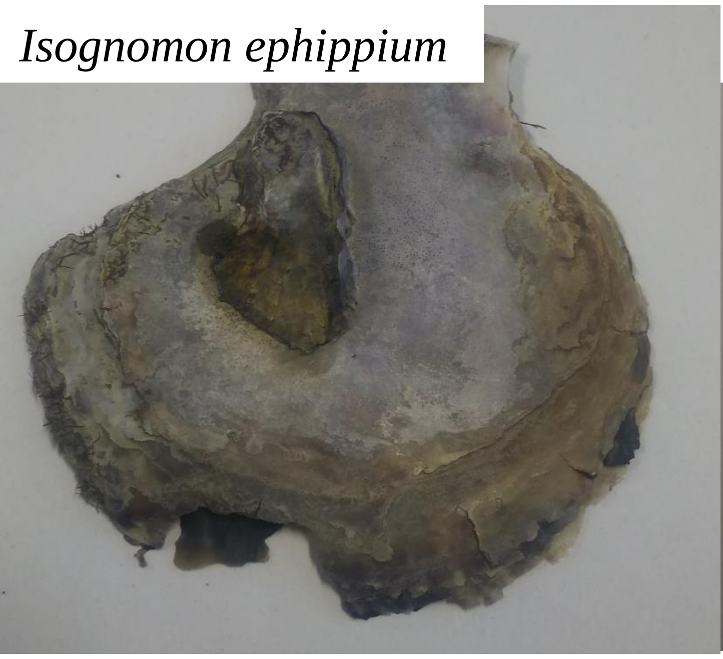

Isognomon ephippium

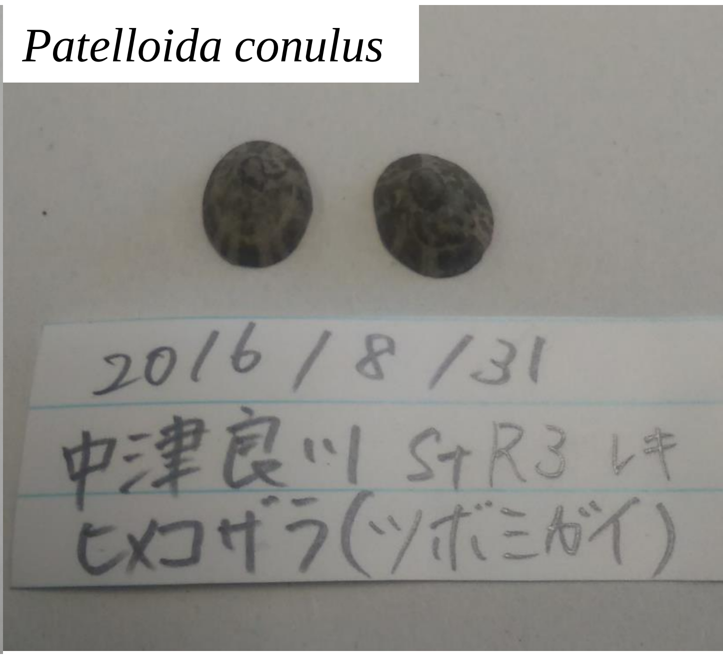

Patelloida conulus

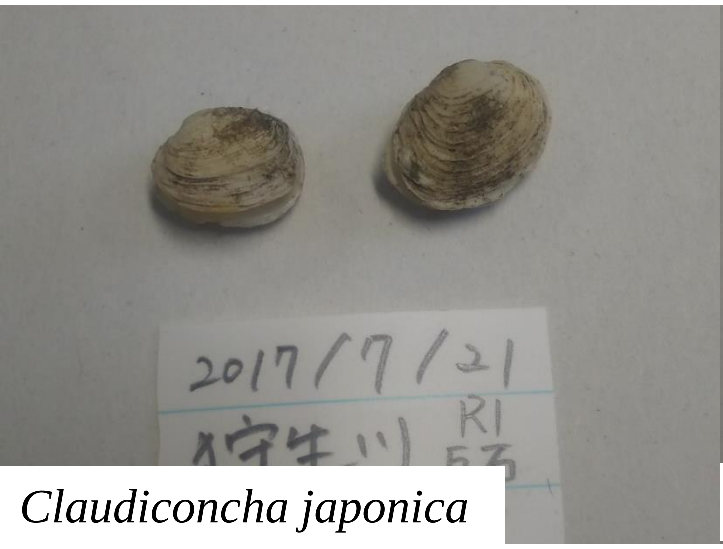

Claudiconcha japonica

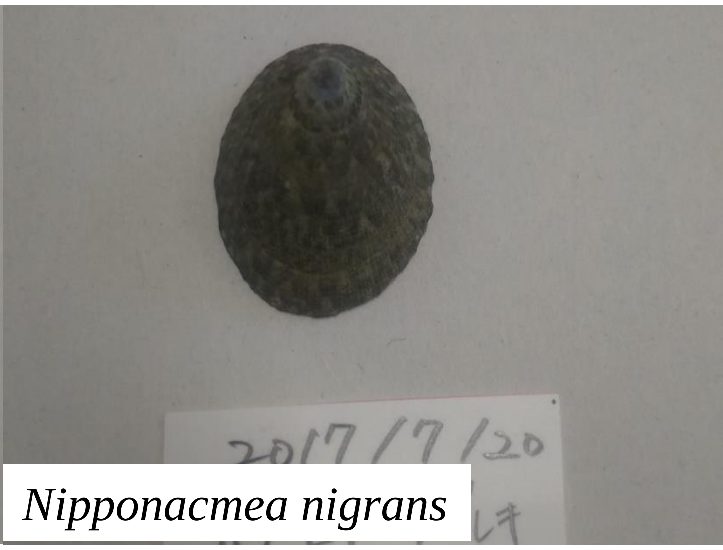

Nipponacmea nigrans

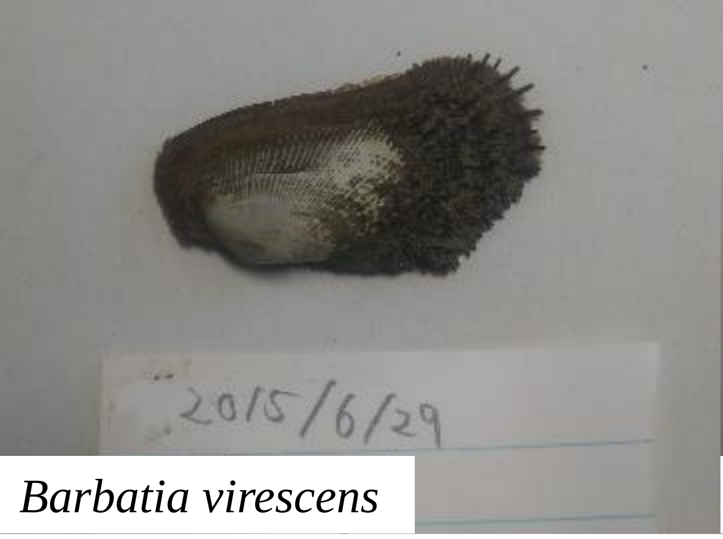

Barbatia virescens

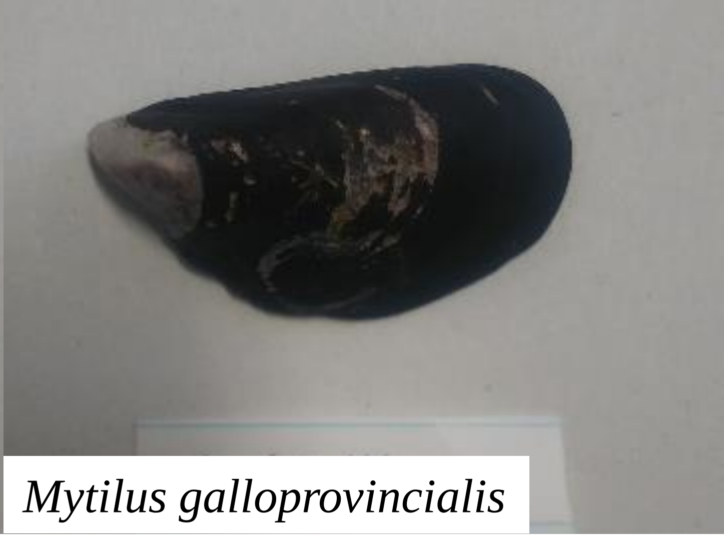

Mytilus galloprovincialis

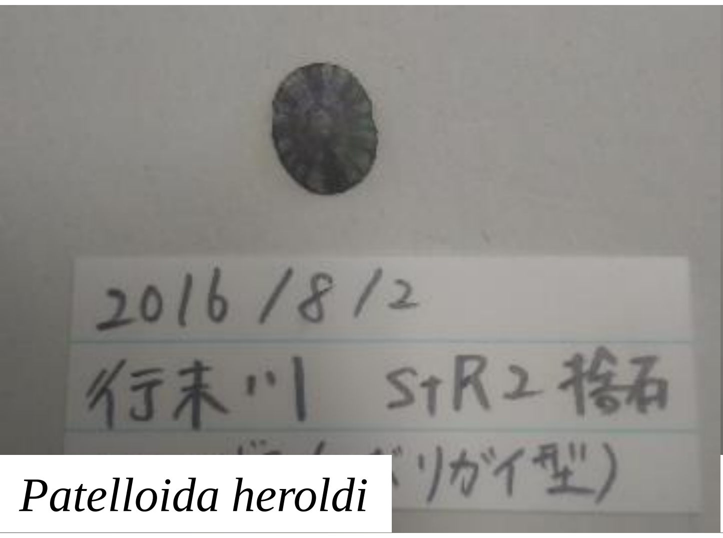

Patelloida heroldi

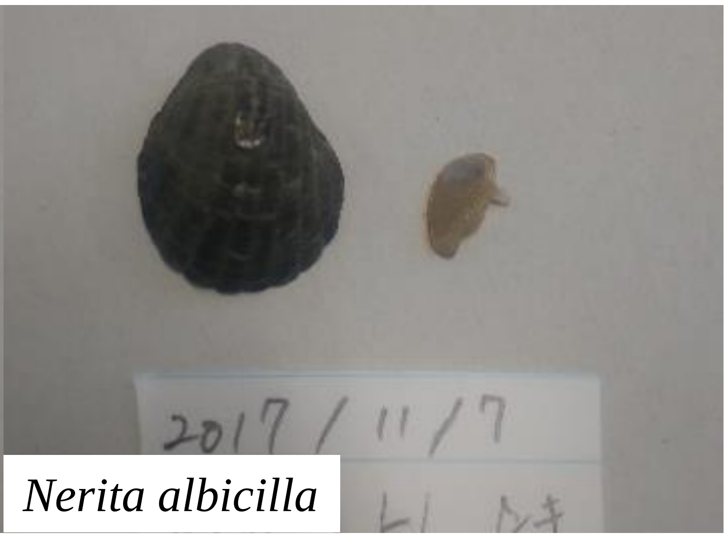

Nerita albicilla

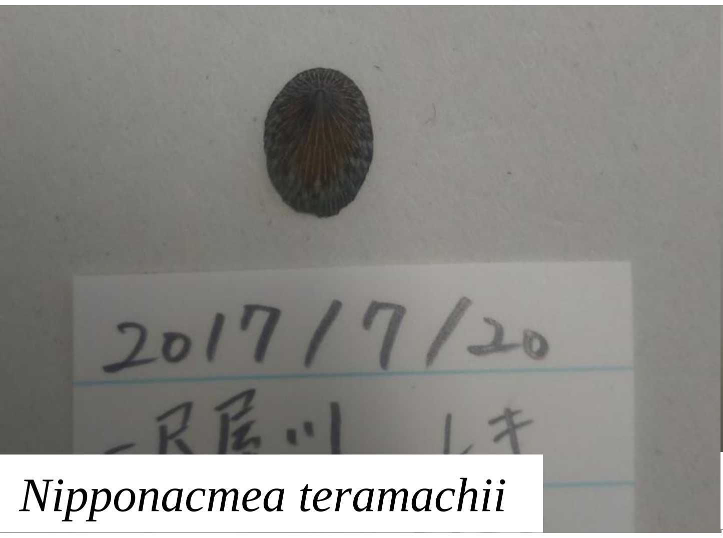

Nipponacmea teramachii

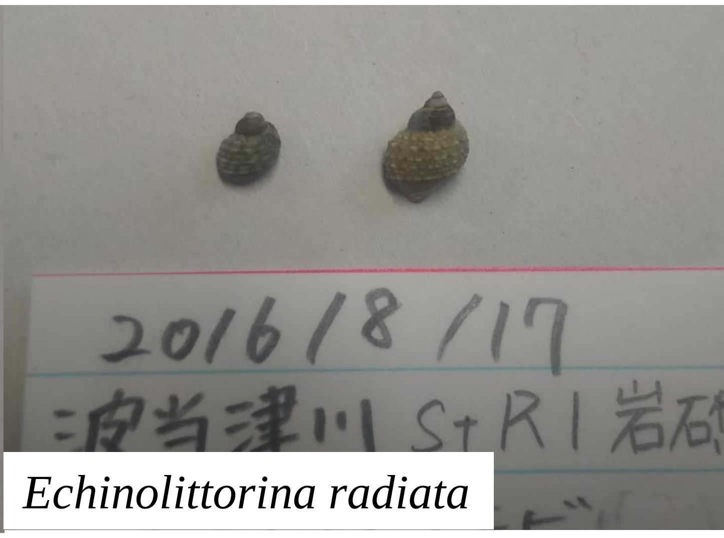

Echinolittorina radiata

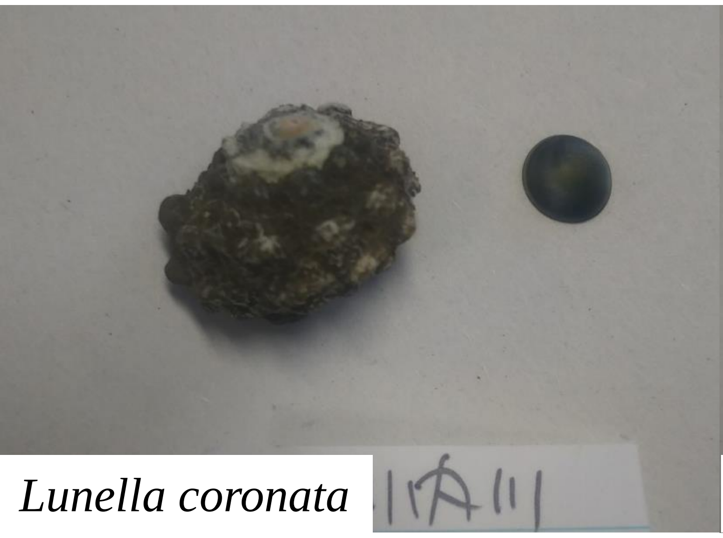

Lunella coronata

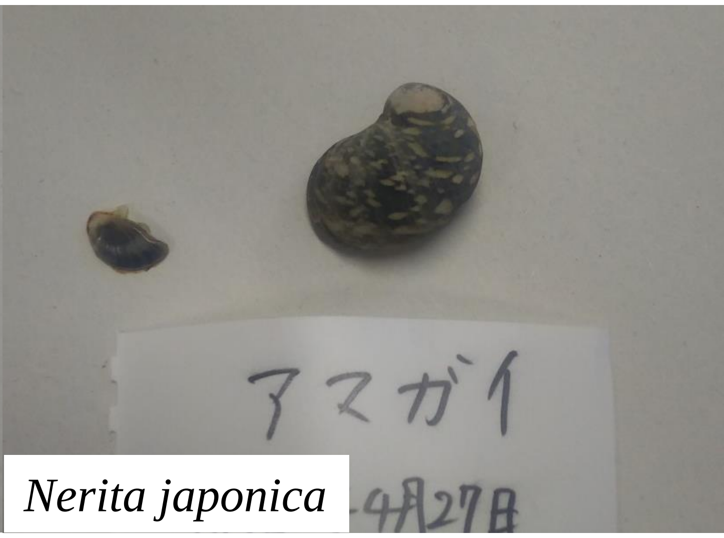

Nerita japonica

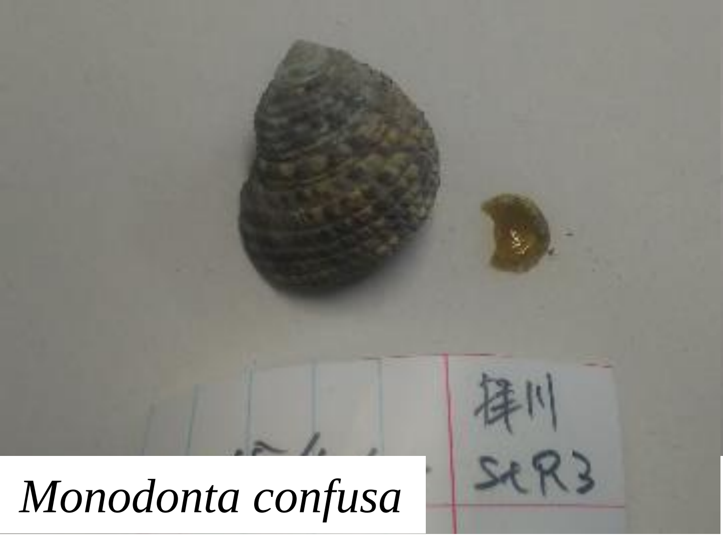
Monodonta confusa

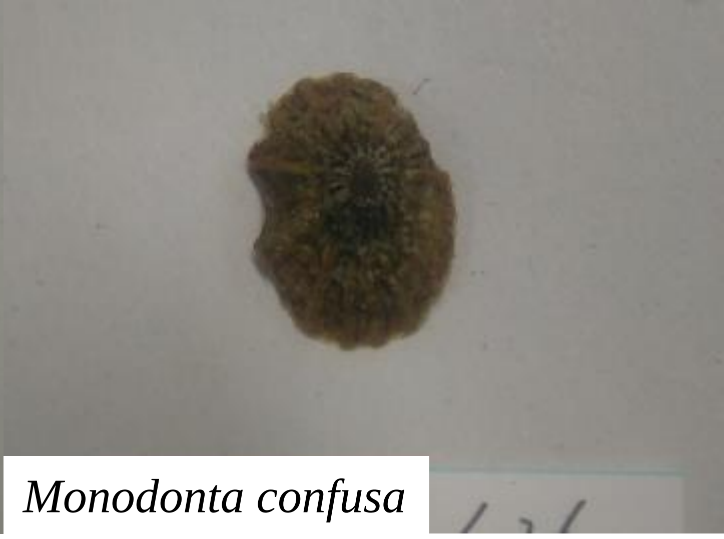
Monodonta confusa

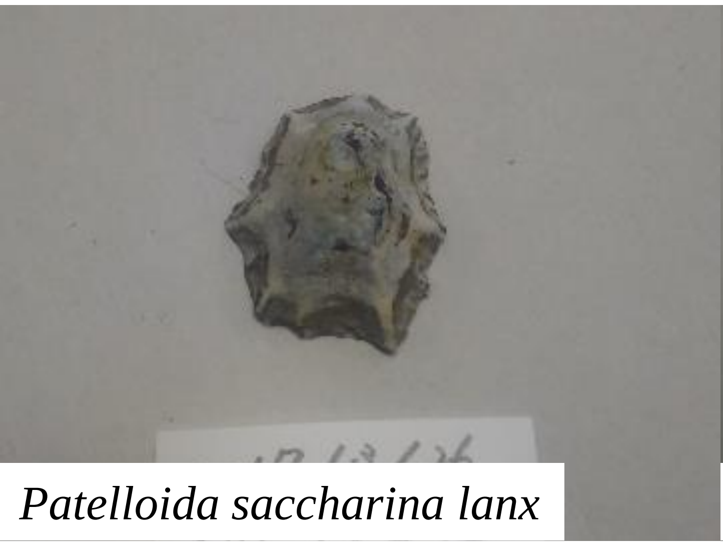
Patelloida saccharina lanx

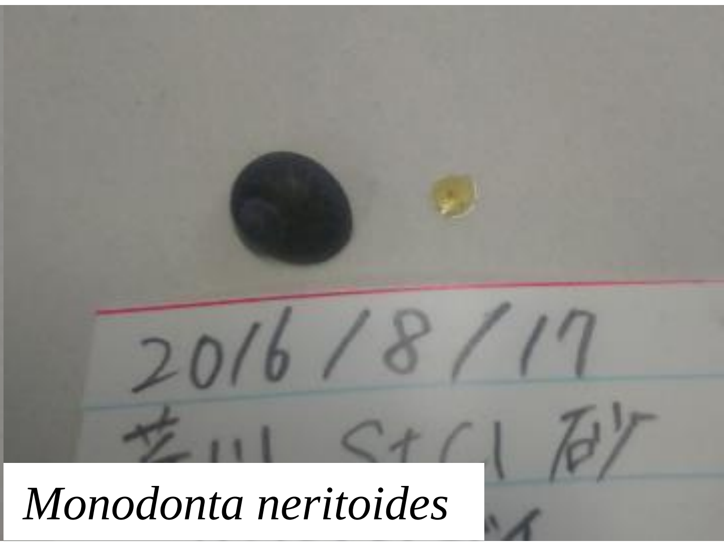

Monodonta neritoides

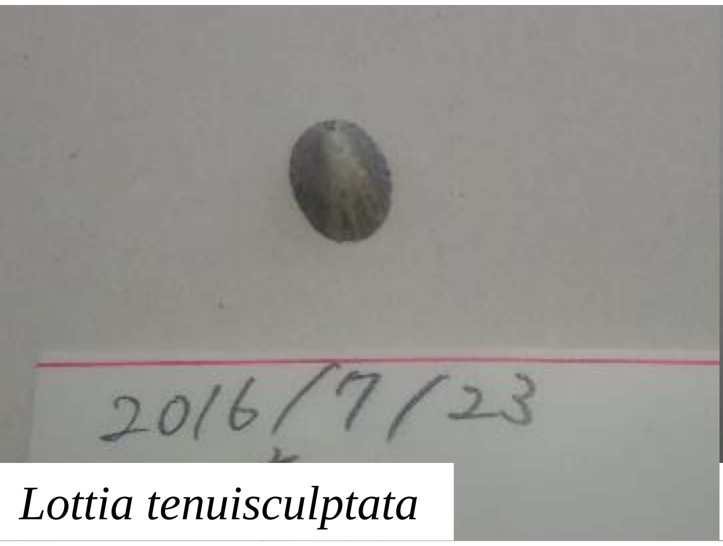

Lottia tenuisculptata

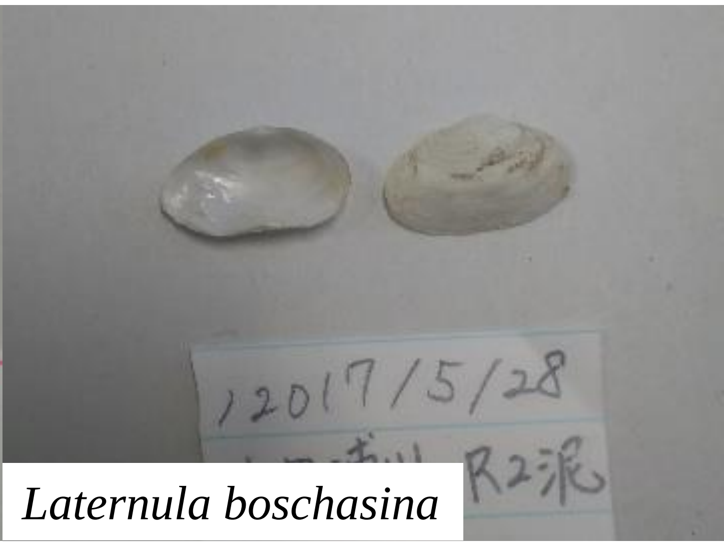

Laternula boschasina

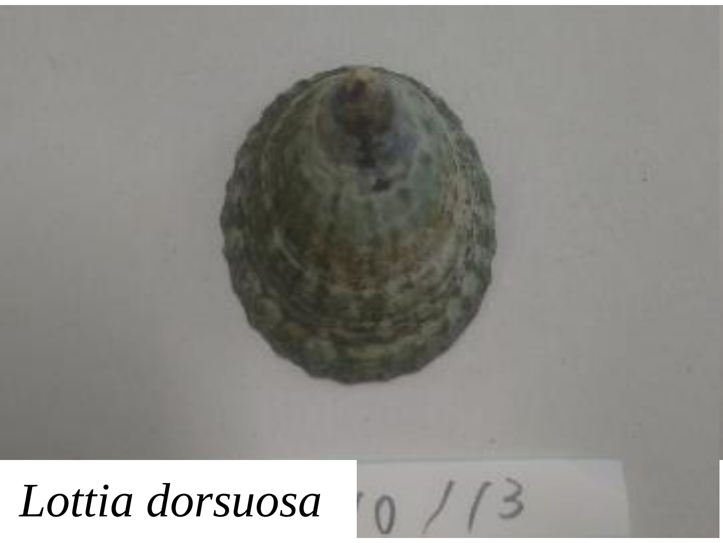
Lottia dorsuosa

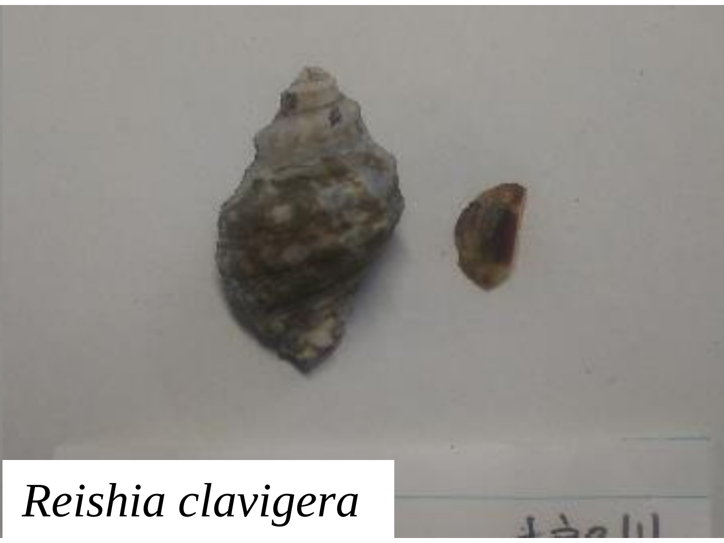
Reishia clavigera

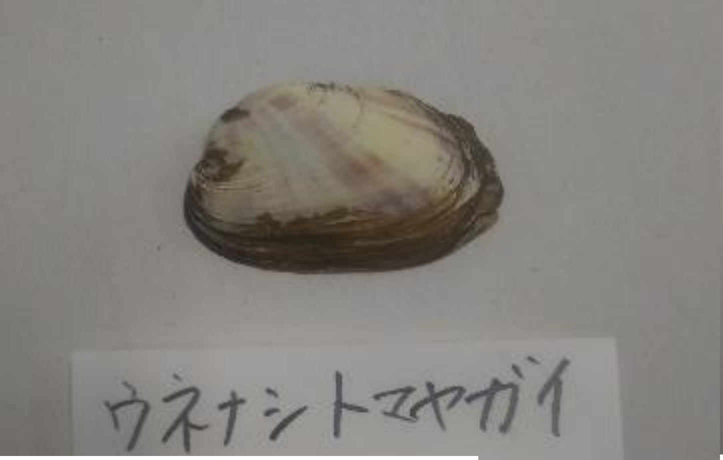

Neotrapezium liratum

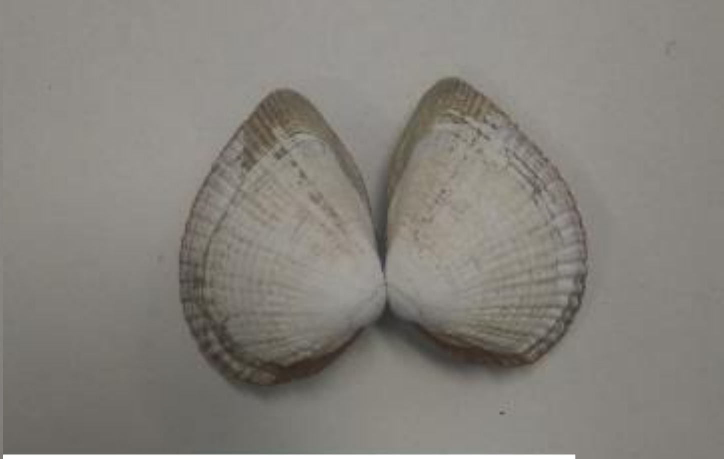

Anomalodiscus squamosus

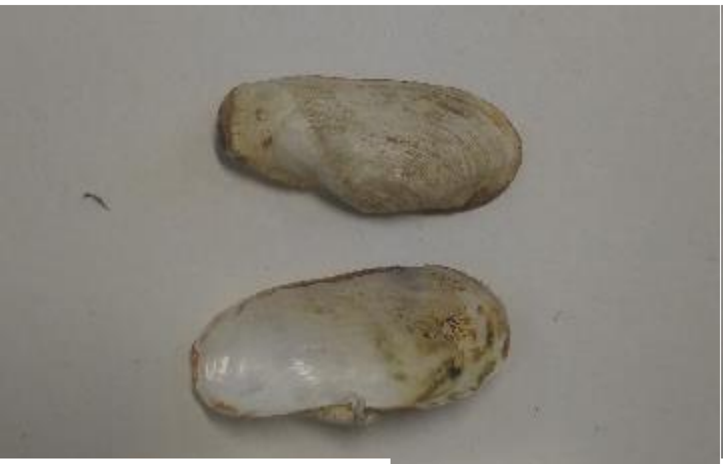

Laternula gracilis

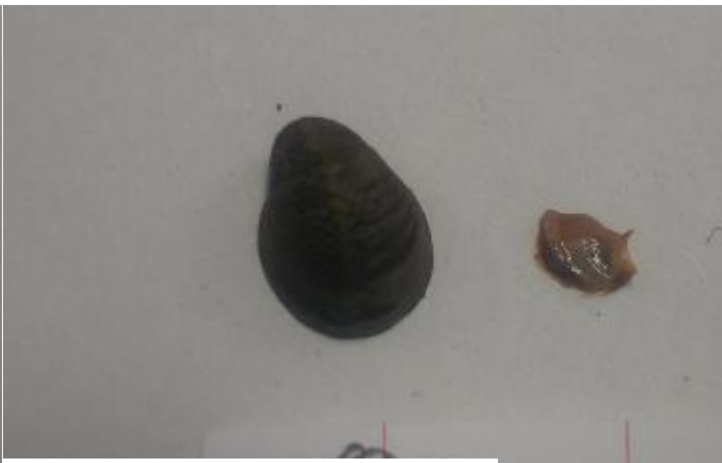

Neripteron cornucopia

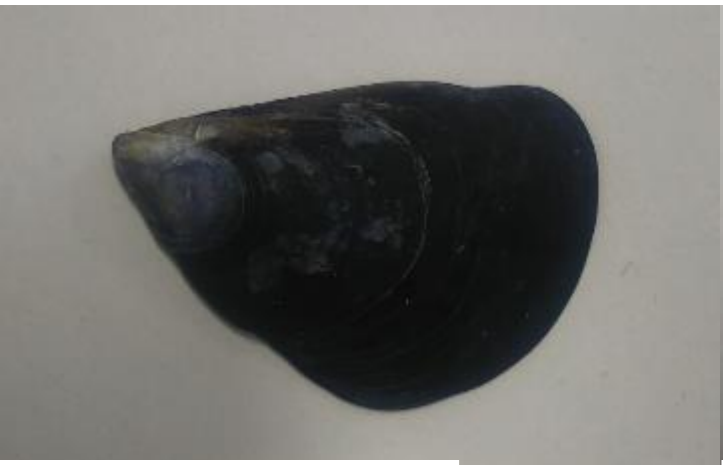

Mytilus unguiculatus

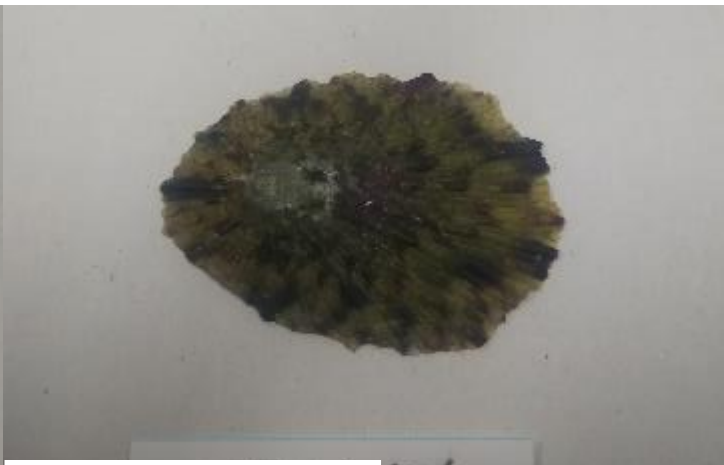

Cellana toreuma

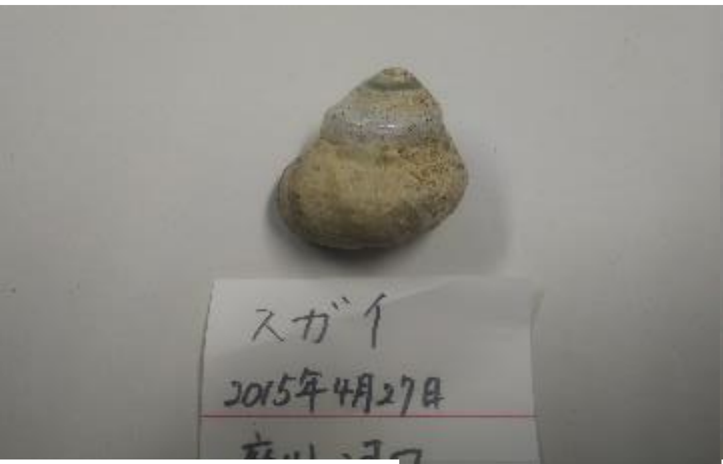

Lunella correensis

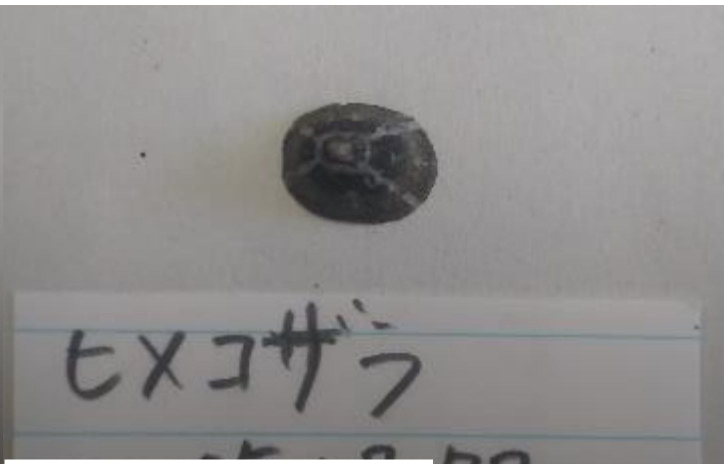

Patelloida pygmaea
